# Plasma β-amyloid in Alzheimer’s disease and vascular disease

**DOI:** 10.1038/srep26801

**Published:** 2016-05-31

**Authors:** Shorena Janelidze, Erik Stomrud, Sebastian Palmqvist, Henrik Zetterberg, Danielle van Westen, Andreas Jeromin, Linan Song, David Hanlon, Cristina A. Tan Hehir, David Baker, Kaj Blennow, Oskar Hansson

**Affiliations:** 1 Clinical Memory Research Unit, Department of Clinical Sciences Malmö, Lund University, Lund, Sweden; 2 Memory Clinic, Skåne University Hospital, Malmö Sweden; 3 Department of Neurology, Skåne University Hospital, Lund, Sweden; 4 Clinical Neurochemistry Laboratory, Institute of Neuroscience and Physiology, Department of Psychiatry and Neurochemistry, the Sahlgrenska Academy at the University of Gothenburg, Mölndal, Sweden; 5 Department of Molecular Neuroscience, UCL Institute of Neurology, Queen Square, London, UK; 6 Department of Clinical Sciences Lund, Diagnostic radiology, Lund University, Lund, Sweden; 7 Imaging and Function, Skåne University Health Care, Lund, Sweden; 8 Quanterix Corporation, 113 Hartwell Avenue, Lexington, MA, USA; 9 Diagnostics and Life Sciences, GE Global Research, Niskayuna, NY, USA; 10 Janssen R&D, Titusville, NJ, USA

## Abstract

Implementation of amyloid biomarkers in clinical practice would be accelerated if such biomarkers could be measured in blood. We analyzed plasma levels of Aβ42 and Aβ40 in a cohort of 719 individuals (the Swedish BioFINDER study), including patients with subjective cognitive decline (SCD), mild cognitive impairment (MCI), Alzheimer’s disease (AD) dementia and cognitively healthy elderly, using a ultrasensitive immunoassay (Simoa platform). There were weak positive correlations between plasma and cerebrospinal fluid (CSF) levels for both Aβ42 and Aβ40, and negative correlations between plasma Aβ42 and neocortical amyloid deposition (measured with PET). Plasma levels of Aβ42 and Aβ40 were reduced in AD dementia compared with all other diagnostic groups. However, during the preclinical or prodromal AD stages (i.e. in amyloid positive controls, SCD and MCI) plasma concentration of Aβ42 was just moderately decreased whereas Aβ40 levels were unchanged. Higher plasma (but not CSF) levels of Aβ were associated with white matter lesions, cerebral microbleeds, hypertension, diabetes and ischemic heart disease. In summary, plasma Aβ is overtly decreased during the dementia stage of AD indicating that prominent changes in Aβ metabolism occur later in the periphery compared to the brain. Further, increased levels of Aβ in plasma are associated with vascular disease.

Recent clinical trials in Alzheimer’s disease (AD) suggested that the success of new disease-modifying treatments critically depends on biomarkers that could reliably detect AD pathology already at prodromal stages^1^. Considerable progress has been made towards developing cerebrospinal fluid (CSF)^2^ and brain imaging^3^ biomarkers of AD. CSF β-amyloid 42 (Aβ42), the 42 amino acid isoform of Aβ, and amyloid positron emission tomography (PET) have been established as the most specific biomarkers of amyloid deposition in the brain^2^. In sporadic AD, CSF Aβ42 is reduced as early as 10–20 years before the onset of clinical symptoms^4^,^5^. Moreover, there are strong inverse correlations between CSF levels of Aβ42 and cortical amyloid PET ligand binding^6^.

The abnormal Aβ status established by either CSF analysis or PET imaging has been incorporated in the diagnostic criteria for AD proposed by both the International Working Group (IWG) for New Research Criteria for the Diagnosis of AD and by the US National Institute on Aging–Alzheimer’s Association (NIA-AA)^7^,^8^. However, for large-scale assessments of patients in primary care settings, blood-based biomarkers are desirable because blood collection is minimally invasive, cost-effective and procedurally simple. Blood-based tests may be used as an initial diagnostic screen for selection of patients to undergo a full diagnostic work-up at the specialist level, including CSF analysis or PET neuroimaging. Nevertheless, efforts to develop blood-derived biomarkers especially those reflecting Aβ pathology have been largely unsuccessful^9^. Cross-sectional studies assessing Aβ42 concentration in the blood of AD patients have produced conflicting results^2^,^9^. Although some evidence from prospective cohorts suggested that high baseline levels of Aβ42 and Aβ40 in plasma were associated with increased risk of future AD, the findings have not been replicated by other reports^10^,^11^,^12^,^13^. In Alzheimer’s Disease Neuroimaging Initiative (ADNI), plasma Aβ fails to differentiate AD patients from control individuals and amyloid-positive from amyloid-negative individuals, although a weak positive relationship between plasma Aβ40/Aβ42 ratio and Aβ ligand retention on PET was observed in *APOE* ε4-negative subjects only^14^. The Australian Imaging Biomarkers and Lifestyle (AIBL) research team have reported that plasma levels of either Aβ40 or Aβ42 do not associate with AD or neocortical Aβ burden^15^. However, the Aβ42/Aβ40 ratio (note inverse ratio as compared to the ADNI results above) was slightly reduced in patients with AD and correlated inversely with amyloid burden as determined by amyloid PET.

The difficulties in getting consistent data could be, at least in part, related to poor performance and insufficient sensitivity of available analytical methods (mostly ELISA-based techniques) for adequate quantification of the minute amounts of Aβ present in peripheral blood^9^. The recently developed ultrasensitive Simoa technology offers improved analytical sensitivity^16^ that makes it suitable for measurements of AD-related biomarkers in serum and plasma^17^. In the present study, we measured plasma levels of Aβ42 and Aβ40 using Simoa assays in a cohort of 719 individuals including patients with subjective cognitive decline (SCD, n = 174), mild cognitive impairment (MCI, n = 214), AD (n = 57) and cognitively healthy elderly (n = 274). We combined plasma measurements with the analysis of CSF samples, amyloid PET, magnetic resonance imaging (MRI) and cognitive assessments in order to establish whether plasma Aβ42 and Aβ40 may be useful biomarkers of AD.

## Materials and Methods

### Study populations

All participants gave written informed consent to participate in the study. Ethical approval was given by the Ethical Committee of Lund University, Lund, Sweden and all the methods were carried out in accordance with the approved guidelines. [^18^F]flutemetamol PET imaging approval was obtained from the Swedish Medicines and Products Agency and the local Radiation Safety Committee at Skåne University Hospital, Sweden.

The study population stemmed from three cohorts from the prospective and longitudinal Swedish BioFINDER study (www.biofinder.se). The first cohort consisted of 274 cognitively normal elderly participants who were recruited from the population-based Malmö Diet Cancer study. Subjects were eligible for inclusion if they 1) were aged ≥60 years old, 2) scored 28–30 points on the Mini-Mental State Examination (MMSE) at the screening visit, 3) did not have cognitive symptoms as evaluated by a physician, 4) were fluent in Swedish, 5) did not fulfill the criteria of MCI or any dementia. The exclusion criteria were 1) presence of significant neurologic or psychiatric disease (e.g. stroke, Parkinson’s disease, multiple sclerosis, major depression), 2) significant systemic illness making it difficult to participate, 3) refusing lumbar puncture and 4) significant alcohol abuse. Data was collected between 2009 and 2014 in accordance with a standardised protocol.

In the second cohort, 388 non-demented patients were enrolled consecutively at three memory outpatient clinics in Sweden. They were referred for assessment of cognitive complaints and evaluated by physicians with special interest in dementia disorders. Patients were included between 2010 and 2014. The inclusion criteria were: 1) referred to the memory clinics because of cognitive impairment; 2) not fulfilling the criteria for dementia; 3) an MMSE score of 24–30 points; 4) age 60–80 years and 5) fluent in Swedish. The exclusion criteria were: 1) cognitive impairment without doubt explained by another condition (other than prodromal dementia); 2) significant systemic illness making it difficult to participate, 3) refusing lumbar puncture and 4) significant alcohol abuse. Classification into SCD and MCI was based on a neuropsychological battery assessing the cognitive domains of verbal ability, visuospatial construction, episodic memory, executive functions and the clinical assessment by a senior neuropsychologist. These criteria resulted in a clinically relevant population where 45% were classified as SCD and 55% as MCI.

In the third cohort, we included 57 patients with AD at baseline, who were recruited consecutively at the Memory Clinic, Skåne University Hospital, Sweden between 2010 and 2014. The patients were assessed by a medical doctor specialized in dementia disorders. All cases met the DSM-IIIR criteria for dementia^18^ as well as the NINCDS-ADRDA criteria for AD^19^. The exclusion criteria were: 1) significant systemic illness making it difficult to participate and 2) significant alcohol abuse.

In all three cohorts, a medical doctor made the diagnosis of hypotension, diabetes and ischemic heart disease. Ischemic heart disease was defined as stable angina, unstable angina, and myocardial infarction. Thiazide diuretics, calcium channel blockers, ACE inhibitors, angiotensin II receptor antagonists, and beta blockers were categorized as anti-hypertensive/cardio-protective medications.

### Plasma and CSF sampling and analysis

Blood and CSF samples were collected on the same day and at the same time of day (plasma was obtained within 15 min of CSF sampling). For plasma collection, blood was drawn into tubes containing EDTA as anticoagulant. After centrifugation (2000 g, +4 °C, 10 min), plasma samples were aliquoted into polypropylene tubes and stored at −80 °C pending biochemical analyses. Lumbar CSF samples were collected according to a standardized protocol^2^,^20^. CSF samples were centrifuged (2000 g, +4 °C, 10 min) after collection and aliquoted into polypropylene tubes followed by storage at −80 °C. The analysis of CSF followed the Alzheimer’s Association Flow Chart for CSF biomarkers^2^.

Plasma Aβ42 and Aβ40 were analyzed using ultrasensitive Simoa immunoassay (Quanterix, Lexington, MA, USA). In traditional sandwich ELISA of complex matrices such as plasma and serum, issues with spike recovery and lack of dilutional linearity (suggesting matrix interferences) have been reported^21^,^22^. The ultrasensitivity of the Simoa assays, allowing dilution of the plasma/serum samples at 1:4 minimizes these matrix effects. The overall benefits of the Simoa assays are not only its high sensitivity and precision, but also the elimination of matrix interferences. The Simoa Aβ40 and Aβ42 assays both utilize the same capture antibody targeting the N-terminus of β-amyloid and different C-terminus detection antibodies specific to Aβ40 and Aβ42. The Aβ40 assay uses β-amyloid (1–40) peptide from AnaSpec (AnaSpec, Fremont, CA, USA) as standard and the Aβ42 assay uses the β-amyloid (1–42) peptide from Covance (Covance Inc., Princeton, NJ, USA) as standard. For each assay, capture antibody was first covalently conjugated to magnetic particles utilizing a standard EDC coupling procedure and detection antibody was biotinylated. In the first step of the assay, antibody coated paramagnetic capture beads, biotinylated detection antibodies, and samples were combined, during which target molecules present in the sample were captured by the capture beads and labeled with the biotinylated detection antibodies. After washing, a conjugate of streptavidin-β-galactosidase (SβG) was mixed with the capture beads where SβG bound to the biotin, resulting in enzyme labeling of captured target molecules. Following a second wash, the capture beads were resuspended in a resorufin β-D-galactopyranoside (RGP) substrate solution and transferred to the Simoa array disc for detection^16^. All samples were diluted 4-fold for Aβ42 and 8-fold for Aβ40 using a proper sample diluent (PBS containing carrier protein and detergent) for measurement. The lower limit of detection (LLoD), defined as a concentration corresponding to a signal level of 2.5 SD above assay background, was 0.019 and 0.16 pg/mL for Simoa Aβ42 and Aβ40 assays, respectively. The lower of limit of quantification was 0.167 pg/ml for Aβ42 (11% dose CV and 90% recovery) and 1.939 pg/mL for Aβ40 (5% dose CV and 99% recovery). Mean spike recoveries of the Simoa Aβ42 and Aβ40 assays were 78.4% and 95.6%, respectively. Both Intra-assay (n = 3) and Inter-assay (n = 13) CVs were less than 10% for both assays. The average CV of measurement of Aβ42 and Aβ40 in all tested plasma samples during this study was 7% and 3%, respectively.

CSF levels of Aβ42 and Aβ40 were analyzed with Euroimmun immunoassay (EUROIMMUN AG, Lübeck, Germany)^23^,^24^ in all study participants. This was done before the analysis of plasma samples using Simoa platform. Because CSF levels of Aβ obtained with Simoa and Euroimmun immunoassays strongly correlated in a subset of 69 of patients, (Aβ42, Pearson’s r = 0.912; Aβ40, Pearson’s r = 0.913; all p < 0.001, Supplementary Fig. S1) we used Euroimmun-derived CSF measurements of Aβ42 and Aβ40 in the present study.

### Brain imaging

#### [^18^F]flutemetamol PET

Three hundred and forty individuals including 125 control subjects, 103 SCD and 112 MCI patients completed [^18^F]flutemetamol PET scans. [^18^F]flutemetamol was manufactured at the radiopharmaceutical production site in Risø, Denmark, using a FASTlab synthesizer module (GE Healthcare, Cleveland, OH). Subjects received a single dose of [^18^F]flutemetamol according to a method described previously^25^. PET/CT scanning of the brain was conducted at two sites using the same type of scanner (Gemini, Philips Healthcare, Best, the Netherlands). Summed PET images from 90–110 min post injection representing the average uptake of [^18^F]flutemetamol over this time were analyzed using NeuroMarQ software (provided by GE Healthcare, Cleveland, OH). A volume of interest (VOI) template was applied for the following 9 bilateral regions: prefrontal, parietal, lateral temporal, medial temporal, sensorimotor, occipital, anterior cingulate, posterior cingulate/precuneus and a global neocortical composite region^26^. The standardized uptake value ratio (SUVR) was defined as the uptake in a VOI normalized for the mean cerebellar cortex uptake.

#### Magnetic Resonance Imaging

A total of 620 individuals underwent MRI imaging including 266 control subjects, 161 SCD and 193 MCI patients. MR imaging was performed at a 3 T Siemens® Trio system equipped with a standard 12 channel head coil. Axial T2 FLAIR (27 slices, voxel size 0.7 × 0.7 × 5.2 mm3), coronal GRE (25 slices, voxel size 0.9 × 0.9 × 6.5 mm3) and coronal MPRAGE (180 slices, voxel size 1 × 1 × 1.2 mm3) images were acquired. Visual rating of WML was performed according to the ARWMC scale (0–30 points)^27^. For statistical analysis, scores from the left and right hemispheres were summarized. The presence of cerebral microbleeds (CBM) was rated according to the MARS scale. This variable was dichotomized as CMB in any hemisphere, present or non-present^28^.

### Statistical analyses

SPSS (IBM, Armonk, NY, US) was used for statistical analysis. Associations between plasma and CSF Aβ as well as between plasma Aβ and composite [^18^F]flutemetamol SUVR in each diagnostic group and in the total sample were first evaluated with Pearson’s correlation analysis. When significant correlations were found the associations between plasma Aβ, CSF Aβ and composite [^18^F]flutemetamol SUVR were further investigated using reduced major axis regression (RMA). The 95% confidence intervals (CI) for the slope estimates were calculated using bootstraping. RMA and all subsequent statistical analysis were adjusted for age and gender. For comparisons of plasma Aβ levels between the diagnostic groups, we used univariate general linear models. The effects of *APOE* genotype, CBM, hypertension, ischemic heart disease and anti-hypertensive/cardio-protective medications on plasma Aβ levels were assessed with univariate general linear models additionally adjusting for diagnosis (with controls, SCD, MCI and AD as diagnostic categories). To test associations between plasma Aβ and cognitive function (cognitive measures of global function (MMSE) or delayed memory recall (ADAS-cog item 3)) and WML we performed linear regression analysis also adjusting for diagnosis. We categorized the study participants into groups with normal and pathological CSF signature using the CSF Aβ42/Aβ40 ratio cutoff ≤0.1^20^,^23^. ROC curves were used to determine how well plasma Aβ could distinguish individuals with a normal versus pathological CSF signature. Similar analysis was conducted for amyloid PET status using the SUVR cutoff >1.42^20^. When comparing all markers between the diagnostic groups the Bonferroni correction was used to adjust for multiple comparisons.

## Results

Demographic and clinical data for the study participants are shown in Table 1.

### Plasma biomarkers, CSF biomarkers and [^18^F]flutemetamol PET

**Figure 1:**
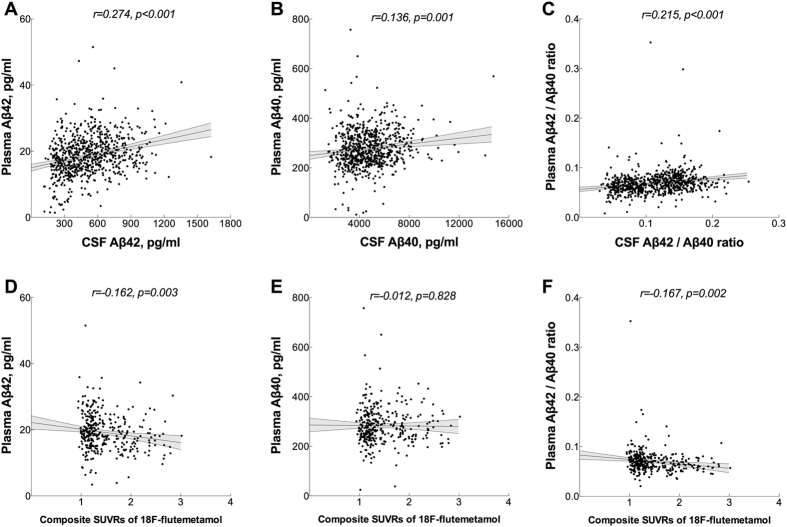
Correlations between plasma and CSF Aβ and between plasma Aβ and [^18^F]flutemetamol SUVR. (**A–C**) Plasma (Simoa immunoassay) and CSF (Euroimmun immunoassay) Aβ42 and Aβ40 were measured in a cohort of 719 individuals (174 SCI, 214 MCI, 57 AD patients and 274 controls). (**D–F**) Neuocortical amyloid deposition was measured using [^18^F]flutemetamol PET in a cohort of 340 individuals (103 SCI, 112 MCI patients and 125 controls). Correlation coefficients (r) and p-values are from Pearson’s correlation analysis. AD, Alzheimer’s disease; CSF, cerebrospinal fluid; SCD, subjective cognitive decline; MCI, mild cognitive impairment; PET, positron emission tomography; SUVR, standardized uptake value ratio.

In order to establish if changes in blood biomarkers are related to AD pathology, we measured Aβ42 and Aβ40 in plasma and CSF samples from cognitively healthy elderly and patients with SCD, MCI and AD. In the total sample, there were weak but significant positive correlations between plasma and CSF levels of Aβ42, Aβ40 and the ratio of Aβ42/Aβ40 (Fig. 1A–C; Table 2). Correlations within individual diagnostic groups are given in Table 2 and shown in Supplementary Fig. S2. The levels for Aβ42 and Aβ42/Aβ40 in CSF and plasma correlated significantly in the control, SCD and MCI groups. In AD patients, there were significant correlations between plasma and CSF levels but only for Aβ42 and Aβ40.

High composite [^18^F]flutemetamol SUVR was associated with lower plasma Aβ42 and lower Aβ42/Aβ40 ratio in the total sample (Fig. 1D–F; Table 3). The correlations with plasma Aβ42 and Aβ40 were significant in the MCI group while the Aβ42/Aβ40 ratio correlated with [^18^F]flutemetamol SUVR in the SCD group (Table 3).

In both plasma and CSF, there were strong correlations between Aβ42 and Aβ40 levels (all r ≥0.511, p < 0.001 in the total samples and individual diagnostic groups; Supplementary Table S1).

The results were similar when the associations between plasma Aβ biomarkers, CSF Aβ biomarkers and [^18^F]flutemetamol SUVR were examined using RMA adjusting for age and gender (95% CIs not containing 0; Tables 1 and 2).

### Plasma and CSF Aβ levels and diagnostic groups

**Figure 2:**
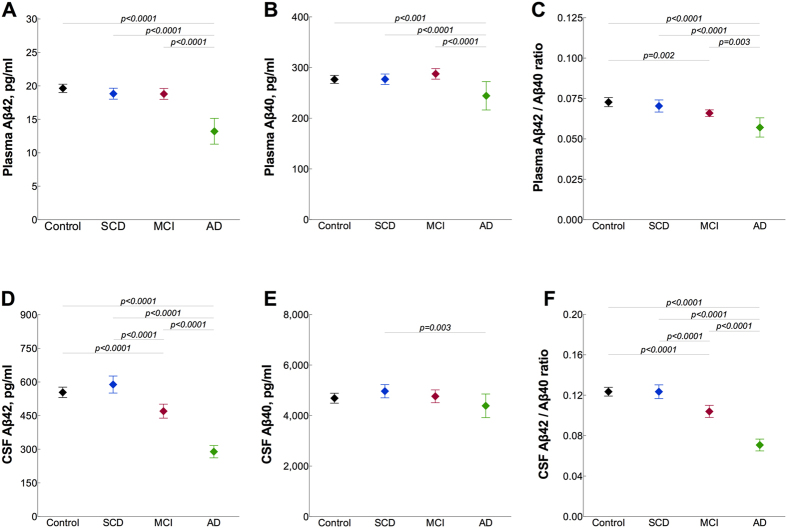
Plasma and CSF Aβ in different diagnostic groups. Plasma (**A–C**, Simoa immunoassay) and CSF (**D–F**, Euroimmun immunoassay) levels of Aβ42, Aβ40 and the Aβ42/Aβ40 ratio in patients with SCD (n = 174), MCI (n = 214), AD (n = 57) and controls (n = 274). Data are presented as mean ± 95% confidence interval (CI); p values are from univariate general linear models controlling for age and gender; statistical significance was set to p < 0.008 (0.05/6) to account for Bonferroni correction. AD, Alzheimer’s disease; CSF, cerebrospinal fluid; SCD, subjective cognitive decline; MCI, mild cognitive impairment.

We next compared the levels of plasma Aβ between the diagnostic groups. Plasma Aβ42 was reduced in AD compared with control, SCD and MCI groups (all p < 0.0001; Fig. 2A; Table 1). However, there were no differences in Aβ42 levels between SCD or MCI patients and controls (Table 1). Plasma levels of Aβ40 were decreased in the AD group compared with controls (p < 0.001), SCD (p < 0.0001) and MCI (p < 0.0001) (Fig. 2B; Table 1). The Aβ42/Aβ40 ratio was lower in the MCI group than in control subjects (p = 0.002) and in AD patients compared with controls (p < 0.0001), SCD (p < 0.0001) and MCI (p = 0.003) (Fig. 2C; Table 1). The levels of Aβ42 and Aβ40 in CSF were found to be in agreement with existing data^6^ (Fig. 2D–F; Table 1).

**Figure 3:**
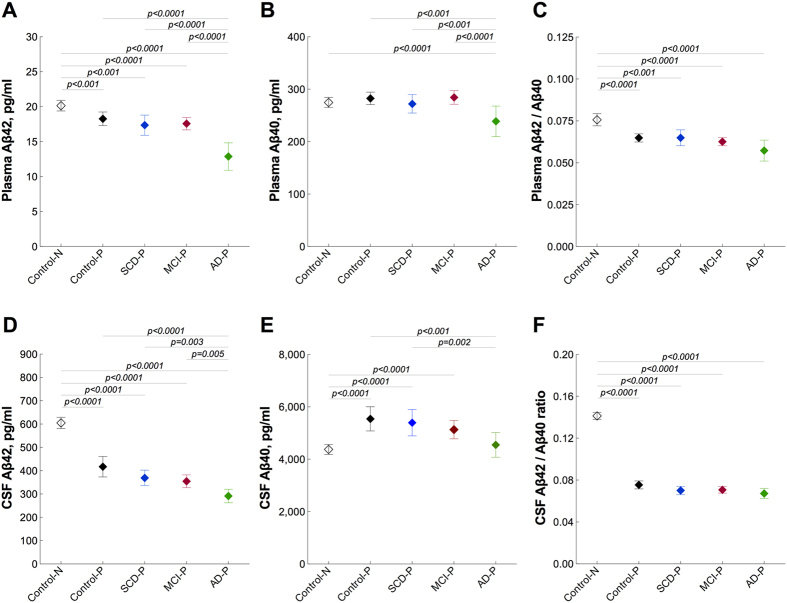
Plasma and CSF Aβ in different diagnostic groups with pathological CSF signature. Plasma (**A–C**, Simoa immunoassay) and CSF **(D–F**, Euroimmun immunoassay) levels of Aβ42, Aβ40 and the Aβ42/Aβ40 ratio in patients with SCD (n = 60), MCI (n = 121), AD (n = 53) and controls (n = 74) with pathological (P) CSF amyloid signature compared to controls with normal (N) CSF amyloid signature (n = 200). Data are presented as mean ± 95% confidence interval (CI); p values are from univariate general linear models controlling for age and gender; statistical significance was set to p < 0.005 (0.05/10) to account for Bonferroni correction. AD, Alzheimer’s disease; CSF, cerebrospinal fluid; SCD, subjective cognitive decline; MCI, mild cognitive impairment.

We also compared diagnostic subgroups with pathological CSF signature (control-P, SCD-P, MCI-P, AD-P with CSF Aβ42/Aβ40 ratio ≤0.1) with control subjects showing normal CSF status (control-N with CSF Aβ42/Aβ40 ratio >0.1). Plasma Aβ42 levels were slightly, but significantly, reduced in control-P (p < 0.001), SCD-P (p < 0.001), MCI-P (p < 0.0001) groups compared with the control-N group (Fig. 3A and Table 4). In the AD-P dementia group, levels were more clearly decreased compared to control-N subject (p < 0.001), and levels were also significantly lower in this group compared to control-P (p < 0.0001), SCD-P (p < 0.0001), MCI-P (p < 0.0001) (Fig. 3A). Plasma Aβ40 was decreased in the AD-P group compared with the control-N (p < 0.0001), control-P (p < 0.001), SCD-P (p < 0.001) and MCI-P (p < 0.0001) groups (Fig. 3B and Table 4). The plasma Aβ42/Aβ40 ratio was reduced in all the diagnostic groups with pathological CSF compared to control individuals with normal CSF (control-P, p < 0.0001; SCD-P, p < 0.001; MCI-P, p < 0.0001; AD-P, p < 0.0001) (Fig. 3C and Table 4). Notably, although the differences in Aβ42 and the Aβ42/Aβ40 ratio between the diagnostic subgroups in plasma were similar to those observed in CSF, they were more pronounced for CSF than for plasma (Fig. 3 and Table 4).

Finally, ROC curve analyses revealed that neither plasma Aβ42 nor the plasma Aβ42/Aβ40 ratio could accurately identify individuals with pathologic CSF signature (Aβ42, AUC = 0.655, 95% CI = 0.615–0.696; the Aβ42/Aβ40 ratio, AUC = 0.683, 95% CI = 0.644–0.722). Furthermore, neither plasma Aβ42 nor the plasma Aβ42/Aβ40 ratio could accurately classify patients with abnormal versus normal PET (Aβ42, AUC = 0.604, 95% CI = 0.543–0.665; the Aβ42/Aβ40 ratio, AUC = 0.621, 95% CI = 0.561–0.682).

### Plasma Aβ and APOE4

Given that individuals with one or two *APOE ε4* alleles have a several fold higher risk for AD and that CSF levels of Aβ42 are affected by *APOE* genotype^29^,^30^,^31^ we assessed the effects of *APOE4* on plasma levels of the Aβ isoforms.

In the total sample, *APOE ε4* carriers showed lower levels of Aβ42 (p < 0.001), Aβ40 (p = 0.009) and lower Aβ42/Aβ40 ratio (p = 0.015) in plasma than non-carriers. When analyzed within individual diagnostic groups, plasma levels of Aβ42 were decreased in *APOE ε4* carriers in controls (p < 0.001) and SCD (p < 0.001), but not in MCI and AD dementia patients. There were no differences in Aβ40 and the Aβ42/Aβ40 ratio between *APOE ε4* carriers and non-carriers in any of the groups.

### Plasma Aβ and cognitive function

We did not find any significant associations between cognitive measures of global function (MMSE) or delayed memory recall (ADAS-cog item 3) and plasma levels of Aβ42 or the plasma Aβ42/Aβ40 ratio when analyzing all individuals simultaneously or when analyzing the different diagnostic groups separately. At the same time, CSF Aβ42 and the CSF Aβ42/Aβ40 ratio correlated with worse delayed memory recall (Aβ42, β = −0.124, p < 0.0001; the Aβ42/Aβ40 ratio, β = −0.137, p < 0.0001).

### Plasma and CSF Aβ and vascular disease

We found that subcortical WML load was weakly associated with increased plasma Aβ42 (β = 0.089, p = 0.023), and Aβ40 (β = 0.093, p = 0.018) and decreased CSF Aβ40 (β = −0.093, p = 0.016). In addition, individuals with CMB (n = 51) showed higher plasma, but not CSF, Aβ42/Aβ40 ratio (p = 0.005) than those without CMB (n = 569).

**Figure 4:**
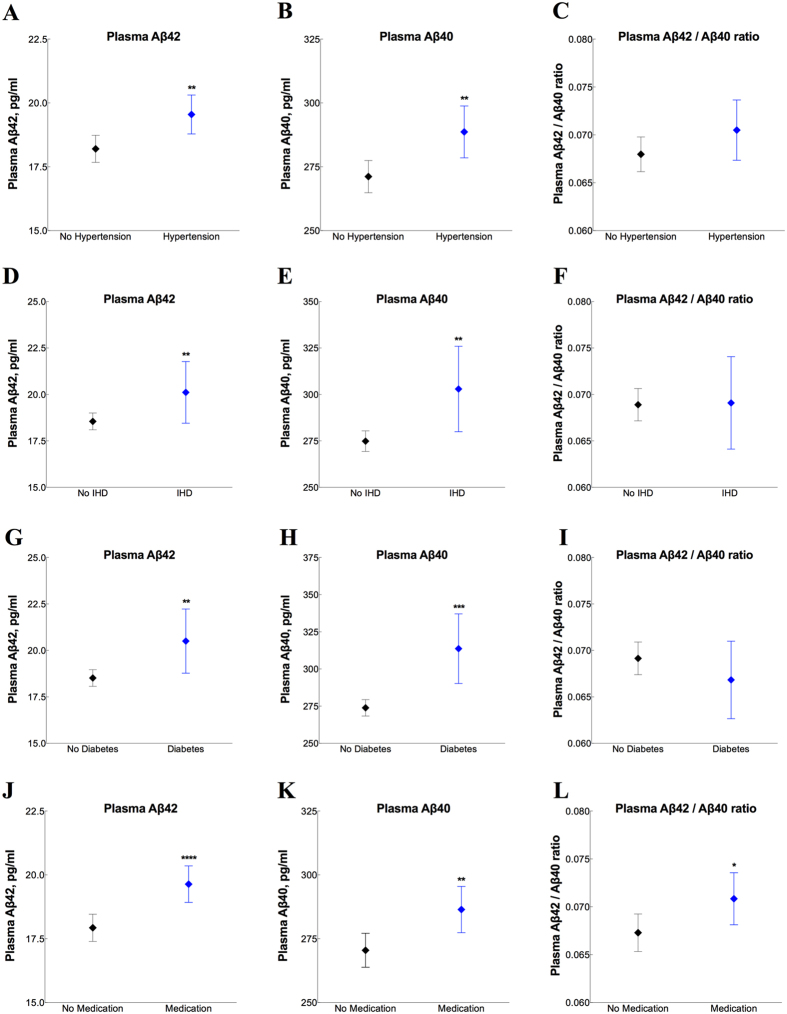
Effects of hypertension, ischemic heart disease and anti-hypertensive/cardio-protective medications on plasma Aβ. Plasma levels of Aβ42, Aβ40 and the Aβ42/Aβ40 ratio (Simoa immunoassay) in patients with and without hypertension (n = 267 and n = 444, respectively), ischemic heart disease (n = 73 and n = 637, respectively), diabetes (n = 69 and n = 641, respectively) or anti-hypertensive/cardio-protective medications (n = 325 and n = 385, respectively). Data are presented as mean ± 95% confidence interval (CI); p values are from univariate general linear models controlling for age, gender and diagnosis; **p < 0.05; **p < 0.01; ***p < 0.001. IHD, ischemic heart disease.

Further, plasma levels of Aβ42 and Aβ40 were increased in subjects with hypertension (Aβ42, p = 0.002; Aβ40, p = 0.002) (Fig. 4A–C), ischemic heart disease (Aβ42, p = 0.050; Aβ40, p = 0.011) (Fig. 4D–F) and diabetes (Aβ42, p = 0.006; Aβ40, p<0.001) (Fig. 4G–I). Plasma Aβ42 and Aβ40 as well as the Aβ42/Aβ40 ratio were also increased in individuals taking anti-hypertensive/cardio-protective medications (Aβ42, p<0.0001; Aβ40, p = 0.006 and the Aβ42/Aβ40 ratio p = 0.016) (Fig. 4J–L). Notably, we did not observe any changes in CSF Aβ42 or Aβ40 in relation to cardiovascular factors (Supplementary Fig. S3).

There were no associations between plasma Aβ and smoking or hyperlipedmia.

## Discussion

In this study, we report that plasma Aβ levels correlate with CSF levels and with Aβ plaque burden in the brain assessed using amyloid PET imaging. We show that plasma Aβ42 and Aβ40 are reduced in AD patients, especially during dementia stages, compared with cognitively healthy control individuals. We also demonstrate increased plasma levels of Aβ were associated with WML, CMB, hypertension, diabetes and ischemic heart disease.

Numerous previous studies investigated plasma levels of Aβ42 and Aβ40 in patients with prodromal AD and AD dementia using conventional ELISA^2^,^9^,^12^,^32^,^33^,^34^. However, because of the inconsistency of the available data, it has been difficult to draw definite conclusions with respect to changes in plasma Aβ concentration in AD. Heterogeneity of sample population, small sample size, confounding factors (in particular age) and insufficient analytical sensitivity for the ELISA methods are all thought to contribute to low reproducibility of the reported results^9^. Here we employed an ultrasensitive digital ELISA to measure plasma Aβ42 and Aβ40. Compared to conventional analog immunoassays, the digital Simoa platform offers improved sensitivity and lower imprecision for the detection of blood proteins^16^. Using the Simoa platform and large cohorts of well-characterized patients and cognitively healthy controls, we found that plasma levels of Aβ42 and Aβ40 are decreased in AD whereas the plasma Aβ42/Aβ40 ratio is decreased in MCI and even more in AD. Although most of the previous investigations showed no differences in plasma Aβ42 between AD patients and cognitively healthy controls^2^,^12^,^35^ our data are in agreement with some studies demonstrating low plasma Aβ42 and/or low plasma Aβ42/Aβ40 ratio in AD^15^,^36^. Further, our results of decreased concentration of plasma Aβ42 in *APOE ε4* carriers compared with non-carriers are also consistent with earlier reports^12^,^37^.

We found reduced levels of Aβ42 and the Aβ42/Aβ40 ratio in plasma of patients with preclinical and prodromal AD (e.g. cognitively healthy individuals, SCD and MCI patients with pathological CSF signature). However, the differences were small in comparison with a marked decline in CSF levels of Aβ42 (Fig. 3) that is observed decades before the onset of clinical symptoms^4^,^5^. These results indicate that AD pathology can be identified in CSF years before overt changes in peripheral blood. Other studies demonstrated that individuals with low plasma Aβ42/Aβ40 ratio (but not plasma Aβ42) at baseline have a somewhat greater risk of future AD and that decrease in plasma Aβ42 levels over time is linked to cognitive decline^10^,^13^. Thus, the low plasma levels of Aβ42 in AD patients could be due to the slow decline along the disease course.

CSF Aβ42 measurements and amyloid PET imaging are increasingly integrated in the clinical work-up of AD as biomarkers of amyloid pathology. However, development of less expensive and less invasive blood biomarkers that could predict CSF Aβ42 and/or amyloid PET status will greatly facilitate widespread implementation of amyloid biomarkers in routine clinical practice. In the present study, we observed significant positive correlations between plasma and CSF concentrations for Aβ42 and the Aβ42/Aβ40 ratio. Moreover, low plasma Aβ42 and Aβ42/Aβ40 ratio were significantly associated with high total brain binding of [^18^F]flutemetamol. Yet similar to previous reports, we found that both associations were relatively weak^12^. Furthermore, neither plasma Aβ42 nor the plasma Aβ42/Aβ40 ratio showed sufficient accuracy to identify individuals with pathologic CSF signature or abnormal amyloid PET. Collectively, our findings suggest that blood Aβ levels reflect only to some extent the dysregulated Aβ metabolism and aggregation in the brain. Other factors that are unrelated to brain amyloid pathology might be modulating the peripheral levels of these peptides. First, Aβ entering peripheral blood may be degraded by circulating enzymes or metabolized in the liver, which would reduce the potential to monitor brain Aβ metabolism. Second, production outside the central nervous system by platelets, skeletal muscle cells and other cells types^38^ probably contributes to the circulating pool of Aβ. Consequently, while cerebral amyloid deposition is accompanied by a considerable decline in CSF Aβ42 levels, the peripheral effects of plaque accumulation in the brain might be more diluted. Notably, we found decreased plasma levels of Aβ40 in AD patients compared with cognitively healthy elderly, whereas in line with available evidence, no change was observed in CSF samples^2^. Altered levels of Aβ have been shown in the skeletal muscle and liver of AD patients^38^ indicating that AD-related changes in the periphery might affect plasma levels of Aβ40 while the CSF levels remain unaltered.

In our study, elevated Aβ42 and Aβ40 in plasma, but not CSF, were associated with WMLs and the plasma, but not CSF, Aβ42/Aβ40 ratio was increased in individuals with CBM. Plasma Aβ42 and Aβ40 have been previously linked to WMLs in non-demented elderly as well as in AD and MCI patients^37^,^39^. Increased plasma levels of Aβ40 have also been described in individuals with infarctions in the ADNI study^12^. There are several potential mechanisms that could explain the association between plasma Aβ and cerebrovascular pathology. Plasma Aβ may affect endothelial cell function and vascular tone thereby leading to cerebral hypoperfusion that eventually results in WMLs^40^. Alternatively, reduced cerebral blood flow, which is an early clinical feature of AD^41^ could promote overproduction of Aβ in endothelial cells and its secretion into the circulation^42^. Increased production of Aβ with marked increases in plasma levels are in fact found after severe ischemia due to cardiac arrest in patients that are resuscitated^43^. In this context it would be of interest to establish if vascular amyloid deposition in cerebral amyloid angiopathy is accompanied by altered blood levels of Aβ42 and Aβ40. Studies in small patient groups have so far produced inconclusive results and warranty future investigations^44^,^45^,^46^. Our finding also indicate that increased Aβ42 and Aβ40 in plasma, but not CSF, are associated with hypertension, diabetes and ischemic heart disease, conditions that adversely impact the function of the vascular system. This is in agreement with previous studies reporting association between plasma Aβ and hypertension^12^,^47^. Further, high plasma Aβ40 has been recently linked to increased cardiovascular mortality in patients with coronary heart disease^48^.

In conclusion, we demonstrate that elevated plasma Aβ is associated with vascular disease both in the brain and in the periphery. In AD, plasma Aβ42 and Aβ40 are markedly reduced during the dementia stages, which is in contrast to the CSF where there is a clear drop in Aβ42, but not Aβ40, already during preclinical stages. However, although low plasma Aβ42 and Aβ42/Aβ40 ratio were associated with amyloid deposition in the brain, these markers did not show diagnostic value in AD. Several panels of plasma AD biomarkers have been recently reported^49^,^50^. Future studies need to determine whether inclusion of plasma Aβ measures might potentially improve the diagnostic performance of the plasma biomarker panels, especially during the dementia stage where we show a clear decrease in plasma Aβ levels.

## Additional Information

**How to cite this article**: Janelidze, S. *et al.* Plasma β-amyloid in Alzheimer’s disease and vascular disease. *Sci. Rep.* **6**, 26801; doi: 10.1038/srep26801 (2016).

## Supplementary Material

Supplementary Information

## Figures and Tables

**Table 1:** Demographic and clinical characteristics of the study participants.

	Control n = 274	SCD n = 174	MCI n = 214	AD n = 57
Gender, % women	61	55	44^a^	60
Age	73 (5)	70 (6)^b^	71 (5)	76 (5)b,c,d
10-word delayed recall (errors)*	2.0 (1.9)	3.4 (2.2) b	6.5 (2.3) b,c	8.5 (1.5) b,c,d
MMSE	29.1 (0.9)	28.4 (1.4) b	27.1 (1.8)b,c	22.1 (3.5) b,c,d
SUVR of [^18^F]flutemetamol	1.3 (0.3)	1.4 (0.4)	1.7 (0.5)b,e	N/A
Plasma Aβ42, *pg/ml*	19.6 (5.2)	18.8 (5.4)	18.8 (6.1)	13.2 (7.3)b,c,d
Plasma Aβ40, *pg/ml*	276.7 (66.1)	276.9 (69.1)	287.6 (77.0)	244.3 (105.8)a,c,d
Plasma Aβ42/Aβ40	0.073 (0.023)	0.070 (0.025)	0.066 (0.015)^f^	0.057 (0.022)b,c,g
CSF Aβ42, *pg/ml*	554.0 (195.1)	588.8 (253.4)	470.1 (232.3)b,c	289.5 (103.8)b,c,d
CSF Aβ40, *pg/ml*	4688.5 (1650.0)	4966.5 (1750.5)	4765.3 (1884.8)	4387.2 (1761.6)^h^
CSF Aβ42/Aβ40	0.123 (0.036)	0.123 (0.045)	0.104 (0.044)b,c	0.070 (0.022)b,c,d

Data are shown as mean (SD) unless otherwise specified.

AD, Alzheimer’s disease; ADAS-cog, Alzheimer’s Disease Assessment Scale-Cognition; MCI, mild cognitive impairment; CSF, cerebrospinal fluid; MMSE, Mini Mental State Examination; SCD, subjective cognitive decline; SUVR, standardized uptake value ratios.

Plasma and CSF Aβ were measured using Simoa and Euroimmun immunoassays, respectively. Demographic factors and clinical characteristics were compared using chi-squared and Mann-Whitney tests. Plasma and CSF biomarkers were analyzed with univariate general linear models controlling for age and gender; statistical significance was set to p < 0.008 to account for Bonferroni correction; ^a^compared with control, p < 0.001; ^b^compared with control, p < 0.0001; ^c^compared with SCD, p < 0.0001; ^d^compared with MCI, p < 0.0001; ^e^compared with SCD, p < 0.001; ^f^compared with control, p = 0.002; ^g^compared with MCI, p = 0.003; ^h^compared with SCD, p = 0.003.

^*^From the ADAS-cog, subtest 3.

**Table 2:** Associations between plasma and CSF Aβ biomarkers.

	All cases	Control	SCD	MCI	AD
Aβ42	**r = 0.274,p < 0.00139 (35, 43)**	**r = 0.188,p = 0.00239 (34, 44)**	**r = 0.182,p = 0.01647 (40, 54)**	**r = 0.270,p < 0.00148 (29, 46)**	**r = 0.288,p = 0.03013 (10, 16)**
Aβ40	**r = 0.136,p = 0.00124 (21, 26)**	r = 0.114,p = 0.059	r = 0.120,p = 0.114	r = 0.083,p = 0.225	**r = 0.349,p = 0.00815 (11, 18)**
Aβ42/Aβ40	**r = 0.215,p < 0.0011.9 (1.4, 2.4)**	**r = 0.166,p = 0.0061.5 (0.6, 2.5)**	**r = 0.160,p = 0.0351.8 (0.8, 2.7)**	**r = 0.202,p = 0.0032.8 (2.4, 3.2)**	r = −0.003,p = 0.981

AD, Alzheimer’s disease; CSF, cerebrospinal fluid; MCI, mild cognitive impairment; SCD, subjective cognitive decline.

Plasma and CSF Aβ were measured using Simoa and Euroimmun immunoassays, respectively. Associations between plasma and CSF Aβ were first evaluated with Pearson’s correlation analysis and if significant correlations were found further investigated using RMA adjusting for age and gender. Data are presented as r, p from Pearson’s correlation analysis and slope estimates (95% CI) from RMA; significant results are shown in bold.

**Table 3:** Associations between plasma Aβ levels and composite amyloid PET SUVR.

	All cases	Control	SCD	MCI
Aβ42	**r = −0.162, p = 0.003−0.08 (−0.09, −0.07)**	r = 0.005,p = 0.953	r = −0.189,p = 0.056	**r = −0.295, p = 0.002−0.09 (−0.1, −0.06)**
Aβ40	r = −0.012, p = 0.828	r = 0.103,p = 0.251	r = 0.014, p = 0.886	**r = −0.199, p = 0.035−0.006 (−0.009, −0.004)**
Aβ42/Aβ40	**r = −0.167, p = 0.002−18 (−27, −10)**	r = −0.130,p = 0.148	**r = −0.205,p = 0.038−33 (−42, −25)**	r = −0.154, p = 0.105

MCI, mild cognitive impairment; SCD, subjective cognitive decline; PET, positron emission tomography; SUVR, standardized uptake value ratio.

Plasma Aβ were measured using Simoa immunoassay. Associations between plasma Aβ and composite [^18^F]flutemetamol SUVR were first evaluated with Pearson’s correlation analysis and if significant correlations were found further investigated using RMA adjusting for age and gender. Data are presented as r, p from Pearson’s correlation analysis and slope estimates (95% CI) from RMA; significant results are shown in bold.

**Table 4:** Plasma and CSF levels of Aβ.

	Control-N n = 200	Control-P n = 74	SCD-P n = 60	MCI-P n = 121	AD-P n = 53
Plasma Aβ42 *pg/ml*	20.1 (5.4)	18.3 (4.2))^a^	17.4 (5.6)^a^	17.6 (4.9)^b^	12.9 (7.1)b,c,d,e
Plasma Aβ40 *pg/ml*	274.6 (70.9)	282.3 (51.3)	271.9 (67.9)	284.3 (72.8)	238.7 (105.5)b,e,f,g
Plasma Aβ42/Aβ40	0.076 (0.026)	0.065 (0.010)^b^	0.065 (0.018)^a^	0.063 (0.013)^b^	0.057 (0.023)^b^
CSF Aβ42 *pg/ml*	604.7 (172.0)	416.9 (189.1)^b^	369.1 (127.7)^b^	354.5 (152.4)^b^	291.3 (105.4)b,c,h,i
CSF Aβ40 *pg/ml*	4373.1 (1378.8)	5540.9 (1997.5)^b^	5395.2 (1949.4)^b^	5129.0 (1937.2)^b^	4548.3 (1712.6)f,j
CSF Aβ42/Aβ40	0.141 (0.023)	0.075 (0.016)^b^	0.070 (0.015)^b^	0.071 (0.017)^b^	0.067 (0.018)^b^

Data are shown as mean (SD).

AD, Alzheimer’s disease; CSF, cerebrospinal fluid; MCI, mild cognitive impairment; SCD, subjective cognitive decline; N, normal CSF signature (CSF Aβ42/Aβ40 ratio > 0.1); P, pathologic CSF signature (CSF Aβ42/Aβ40 ratio ≤0.1).

Plasma and CSF Aβ were measured using Simoa and Euroimmun immunoassays, respectively. Plasma and CSF biomarkers were analyzed with univariate general linear models controlling for age and gender; statistical significance was set to p < 0.005 to account for Bonferroni correction; ^a^compared with control-N, p < 0.001; ^b^compared with control-N, p < 0.0001; ^c^compared with control-P, p < 0.0001; ^d^compared with SCD-P, p < 0.0001; ^e^compared with MCI-P, p < 0.0001; ^f^compared with control-P, p < 0.001; ^g^compared with SCD-P, p < 0.001; ^h^compared with SCD-P, p = 0.003; ^i^compared with MCI-P, p = 0.005; ^j^compared with SCD-P, p = 0.002.
