# Supplementary material for: Plasma β-amyloid in Alzheimer’s disease and vascular disease
Source: Sci Rep. 2016 May 31;6:26801. doi: 10.1038/srep26801 (PMC4886210; doi:10.1038/srep26801)
Supplement: Supplementary Information [file srep26801-s1.doc]

**Supplementary information**

**Plasma β-amyloid in Alzheimer's disease and vascular disease**

Shorena Janelidze, Erik Stomrud, Sebastian Palmqvist, Henrik Zetterberg, Danielle van Westen, Andreas Jeromin, Linan Song, David Hanlon, Cristina A. Tan Hehir, David Baker, Kaj Blennow, Oskar Hansson

**Contents:**

**Supplementary Figure S1.** Correlations between CSF levels of Aβ obtained using Simoa and Euroimmun immunoassays.

**Supplementary Figure S2.** Correlations between plasma and CSF Aβ within diagnostic groups.

**Supplementary Figure S3**. Effects of hypertension, ischemic heart disease and anti-hypertensive/cardio-protective medications on CSF Aβ.

**Supplementary Table S1.** Associations between Aβ42 and Aβ40 in plasma and CSF.

|  | All cases | Control | SCD | MCI | AD |
| --- | --- | --- | --- | --- | --- |
| plasma | **r=0.685, p<0.001** | **r=0.634, p<0.001** | **r=0.611, p<0.001** | **r=0.726, p<0.001** | **r=0.826, p<0.001** |
| CSF | **r=0.529, p<0.001** | **r=0.604, p<0.001** | **r=0.511, p<0.001** | **r=0.518, p<0.001** | **r=0.684, p<0.001** |

AD, Alzheimer's disease; CSF, cerebrospinal fluid; MCI, mild cognitive impairment; SCD, subjective cognitive decline.

Plasma and CSF Aβ were measured using Simoa and Euroimmun immunoassays, respectively. Data are presented as r, p from Pearson's correlation analysis; significant results are shown in bold.

**Supplementary Figure S1.** Correlations between CSF levels of Aβ obtained using Simoa and Euroimmun immunoassays.


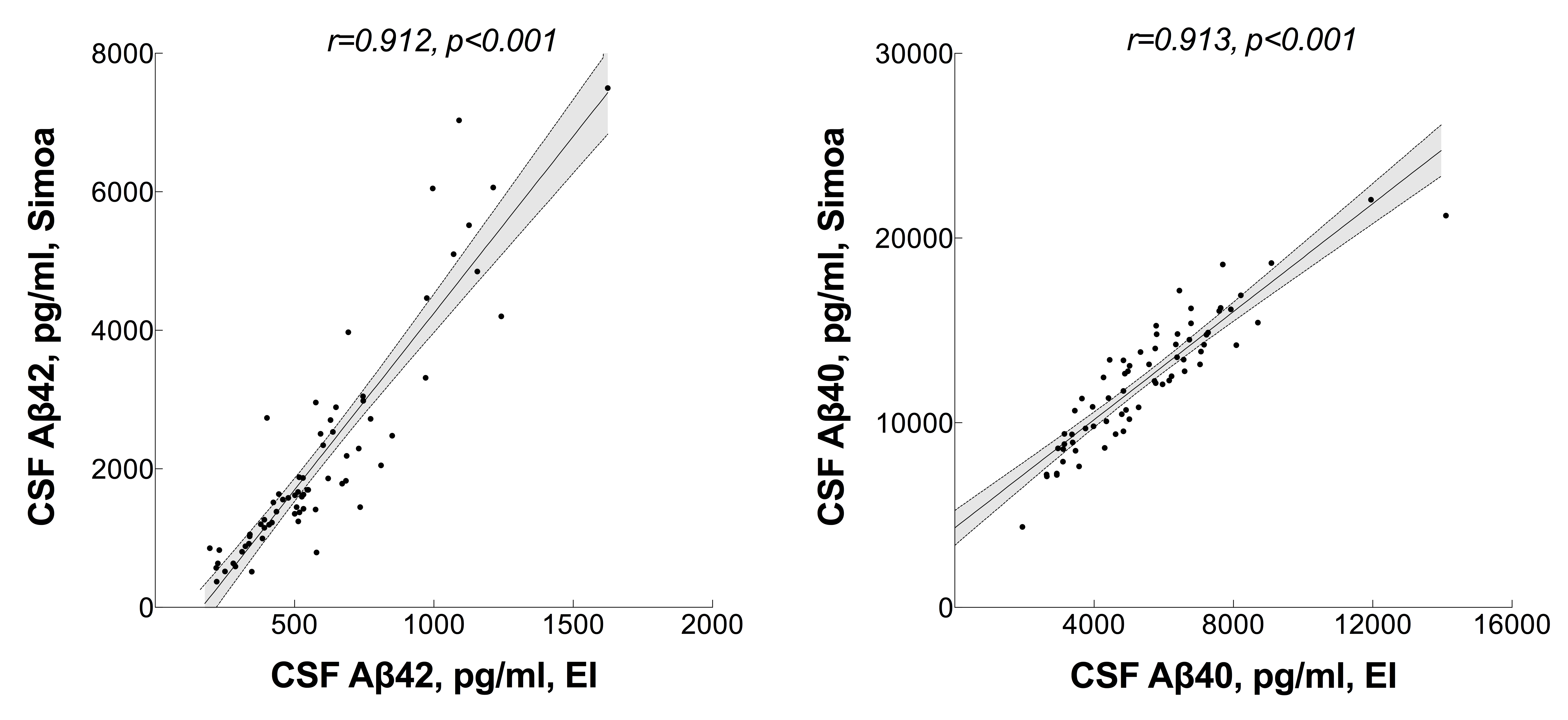


CSF levels of Aβ42 and Aβ40 were measured in a subset of 69 of patients using Simoa and Euroimmun (EI) immunoassays. Correlation coefficients (r) and p-values are from Pearson's correlation analysis. CSF, cerebrospinal fluid; EI, Euroimmun.

**Supplementary Figure S2.** Correlations between plasma and CSF Aβ within diagnostic groups.


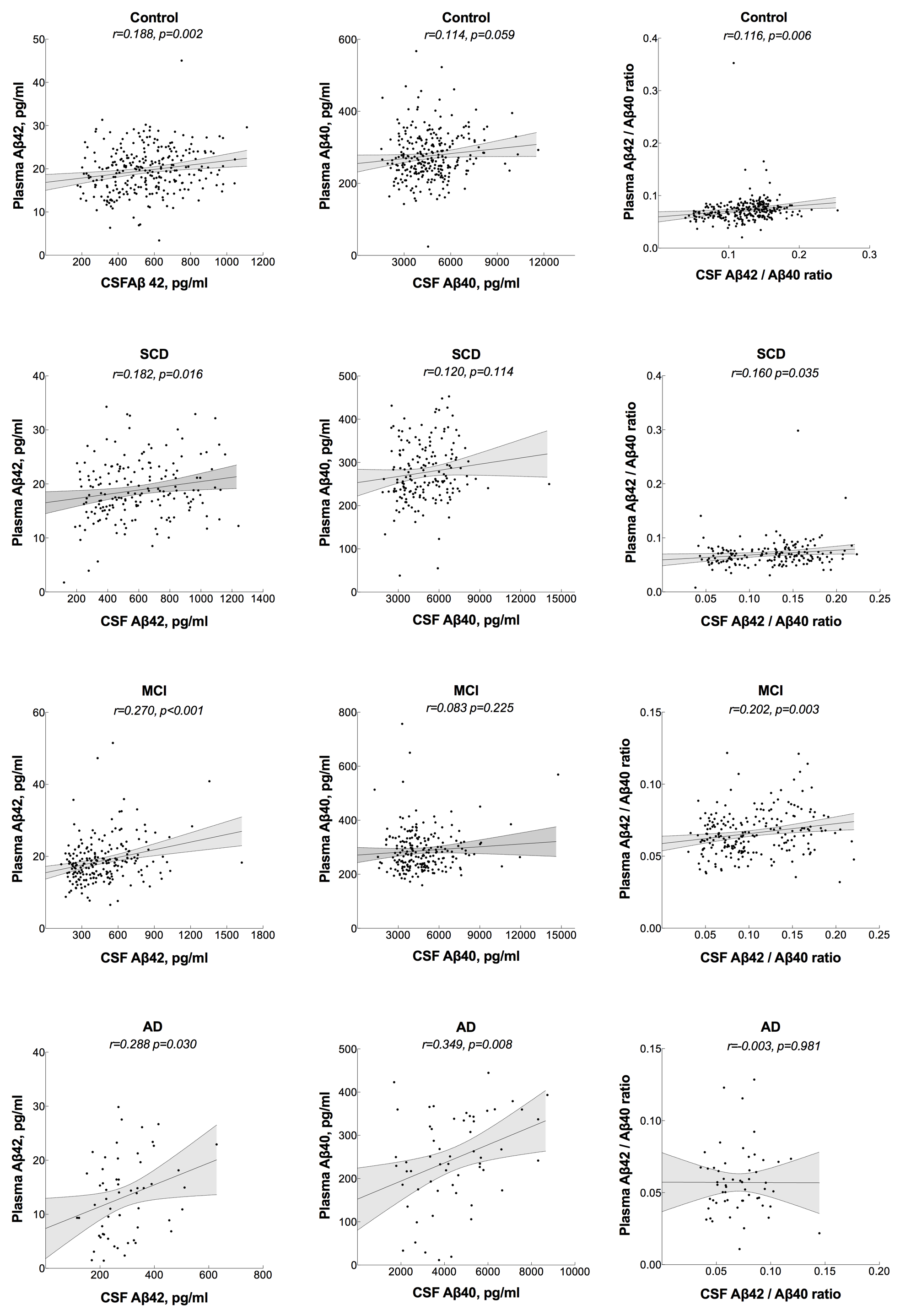


Plasma and CSF Aβ42 and Aβ40 were measured using Simoa and Euroimmun immunoassays, respectively, in 174 SCI, 214 MCI, 57 AD patients and 274 controls. Correlation coefficients (r) and p-values are from Pearson's correlation analysis. AD, Alzheimer's disease; CSF, cerebrospinal fluid; SCD, subjective cognitive decline; MCI, mild cognitive impairment; SUVR, standardized uptake value ratio.

**Supplementary Figure S3.** Effects of hypertension, ischemic heart disease, diabetes and anti-hypertensive/cardio-protective medications on CSF Aβ.


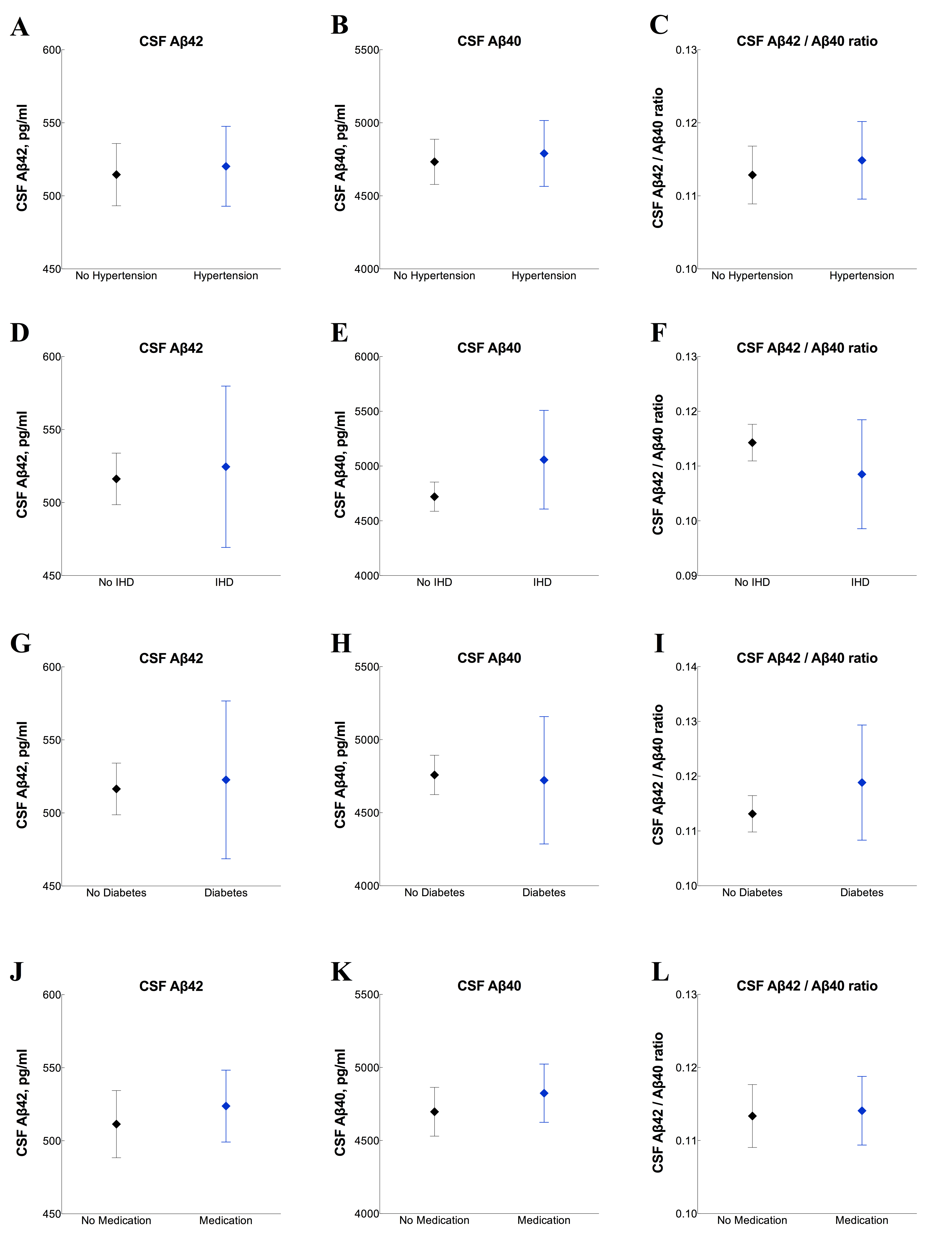


CSF levels of Aβ42 and Aβ40 were measured using Euroimmun immunoassays in patients with and without hypertension (n=267 and n=444, respectively), ischemic heart disease (n=73 and n=637, respectively), diabetes (n=69 and n=641, respectively) or anti-hypertensive/cardio-protective medications (n=325 and n=385, respectively). Data are presented as mean±95% confidence interval (CI); p values are from univariate general linear models controlling for age, gender and diagnosis. CSF, cerebrospinal fluid; IHD, ischemic heart disease.
